# Identification of a novel deletion mutation in *DPY19L2* from an infertile patient with globozoospermia: a case report

**DOI:** 10.1186/s13039-020-00495-1

**Published:** 2020-06-22

**Authors:** You-zhu Li, Rong-feng Wu, Xing-shen Zhu, Wen-sheng Liu, Yuan-yuan Ye, Zhong-xian Lu, Na Li

**Affiliations:** 1 Reproductive Medicine Center, The First Affiliated Hospital of Xiamen University, No. 6 Guchengxi Road, Si Ming, Xiamen, 361003 China; 2 School of Pharmaceutical Sciences, Xiamen University, Xiamen, 361005 Fujian China; 3 Intensive Care Unit, Fujian Medical University Xiamen Humanity Hospital, No.3777 Xianyue Road, Huli, Xiamen, 361009 China

**Keywords:** Case report, Globozoospermia, Gene mutations, CNV sequencing, *DPY19L2*

## Abstract

**Background:**

Male infertility is an increasing medical concern worldwide. In most cases, genetic factors are considered as the main cause of the disease. Globozoospermia (MIM102530) (also known as round-headed sperm) is a rare and severe malformed spermatospermia caused by acrosome deficiency or severe malformation. A subset of genetic mutations, such as *DNAH6, SPATA16, DPY19L2, PICK1,* and *CCIN* related to globozoospermia, have been reported in the past few years. The *DPY19L2* mutation is commonly found in patients with globozoospermia. Herein, a 180-kbp homozygote deletion at 12q14.2 (g.63950001–64130000) was identified by copy number variation sequencing (CNVseq) in a patient with a globozoospermia, including the complete deletion of *DPY19L2*.

**Case presentation:**

A 27-year-old patient at the First Affiliated Hospital of Xiamen University was diagnosed with infertility because, despite normal sexual activity for 4 years, his wife did not conceive. The patient was in good health with no obvious discomfort, no history of adverse chemical exposure, and no vices, such as smoking and drinking. The physical examination revealed normal genital development. However, semen tests showed a normal sperm count of 0% and the morphology was the round head. Sperm cytology showed that acrosomal enzyme was lower than normal. Reproductive hormones were in the normal range. B ultrasound did not show any abnormal seminal vesicle, prostate, bilateral testis, epididymis, and spermatic veins. The karyotype was normal, 46, XY, and no microdeletion of Y chromosome was detected. However, a homozygous deletion mutation was found in *DPY19L2*, which was further diagnosed as globozoospermia.

**Conclusions:**

The present study reported a male infertility patient who was diagnosed with globozoospermia. The analysis of gene mutations revealed that *DPY19L2* had a homozygous mutation, which was the primary cause of globozoospermia.

## Background

Infertility has gradually become a medical issue attracting worldwide attention. In most cases, the gene factors are considered to be a major cause of the disease [1, 2]. Globozoospermia (MIM102530) is a rare (incidence 0.1%) form of severe monomorphic teratozoospermia that leads to primary male infertility and is characterized by round-headed spermatozoa without acrosome, an abnormal nuclear membrane, and midpiece defects in the ejaculate. Thus, globozoospermia is a severe reproductive issue requiring urgent resolution [3–6].

Previous studies have suggested that gene mutations might be the pathology underlying human globozoospermia, and several genes, such as *DNAH6* [7] and *SPATA16* [8], cause globozoospermia. *DPY19L2* (DPY-19-like 2, MIM 613893) mutations affect sperm head elongation and acrosome formation and accounts for approximately 75% of the patients with globozoospermia [9–13]. *SPATA16* was the first gene reported to be involved in the pathogenesis of globozoospermia in humans [8]. SPATA16 are located in Golgi body and anterior acrosome vesicles that are transported to the anterior segment to form acrosome during the formation of the spermatozoon, suggesting a key role of the protein in the formation of acrosome [8, 14]. *SPATA16* mutations have been identified in some acrosome absent cases that result in globozoospermia as the membrane is unable to bind the zona pellucida and fertilize the oocyte [8, 14, 15]. *PICK1* gene encoding a cytosolic protein is found in the proacrosomal vesicles of round spermatids; the mutations in this gene lead to the failure of the merge of proacrosomal vesicles, thereby resulting in globozoospermia [16–18]. *CCIN* encodes a major basic protein of the mammalian sperm head cytoskeleton, and its absence or altered arrangement of the calicin protein is related to globozoospermia [19, 20]. In mouse models, the knockout of *Gopc* [21], *Hrb* [22, 23], *Csnk2a2* [24], *Hsp90beta1* [25], *Vps54* [26], *Mfsd14a* [27], and *GM130* [28] can replicate the round-headed sperm phenotype, but whether these gene mutations also cause this phenotype in humans needs to be investigated further. Although the mutations of *DPY19L2* account for up to 75% of globozoospermia, the genetic mutation spectrum is not yet completely fully elucidated, necessitating an in-depth investigation to deduce the involvement of *DPY19L2* in globozoospermia.

In the present study, we investigated an infertile patient with globozoospermia and identified a homozygous deletion mutation in *DPY19L2*. This study confirmed that *DPY19L2* mutation is the main cause of globozoospermia, which broadened the mutation spectrum of the gene.

## Case presentation

### Clinical presentation and family history

**Fig. 1:**
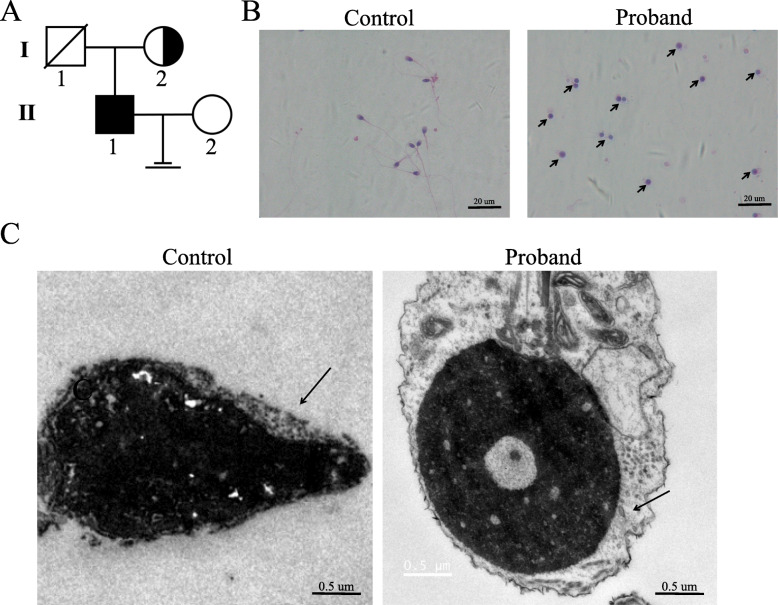
Consanguineous family analysis and phenotypes of patients with globozoospermia. **a**. **Consanguineous pedigree of the proband family with complete deletion of*DPY19L2*.** The patient (II:1) was suffering from globozoospermia had homozygous deletion of exons 1, 11 and 22 of *DPY19L2*. And heterozygous deletion in exons 1, 11, 22 of *DPY19L2* in mother of proband (I:2), while no mutation was found in exons 1, 11 and 22 of *DPY19L2* of his father (I:1). **b**. **Papanicolaou staining of sperm cells from normal control and the proband globozoospermia.** Black arrows indicate spermatozoa from globozoospermia. Scale bar: 20 μm. **c**. **Ultrastructure of the Sperm from normal control and patient with complete loss of DPY19L2** showing that the DPY19L2-null spermatozoa had round sperm head. The black arrow indicates the head of normal control sperm and the head of DPY19L2 completely missing sperm.

The proband (aged 27 years, II:1) and his family were recruited from the First Affiliated Hospital of Xiamen University. Pedigree analysis revealed recessive autosomal (AR) inheritance (Fig. 1a). He had sexual life 2–3 times/ week with normal erection and ejaculation during the last 4 years after marriage, but his wife did not conceive. The patient did not have any bad chemical contact history or habits such as smoking and drinking. The patient was in good health with no obvious discomfort. Physical examination data were as follows: height, 174 cm; weight, 66 kg; external genital development, normal; bilateral testicular size and bilateral spermatic vein, normal. The semen was light yellow and could be liquefied within 30 min. The examination results from our hospital were as follows: semen volume, 5.5–6.0 mL; semen pH, 7.2; sperm density, 9.1–11.3 × 10^6^/mL; prorsad percentage motility, 15–18%; non-prorsad percentage motility, 12–15%. Sperm morphology examined by Papanicolaou staining showed 0% normal morphology. The biochemical testing of seminal plasma showed that the content of sperm acrosomase was 32.6 uIU/10 × ^6^; neutral glycosidase, 20.6 mU; fructose level, 17.5 μmol; zinc level, 5.4 μmol. The reproductive hormones were within normal ranges (follicle-stimulating hormone (FSH) 4.56 mIU/mL, luteinizing hormone (LH) 5.87 mIU/mL, T 4.34 ng/mL, E2 33 pg/mL, PRL 12.62 ng/mL). B-ultrasound showed no abnormalities in the seminal vesicles, prostate, bilateral testes, epididymis, and spermatic veins. The chromosomal karyotype of the patient was normal, 46, XY, and no microdeletions were detected in the Y chromosome. The patient’s family had yet to undergo follow-up treatment.

A volume of 5 mL peripheral blood was withdrawn from the patient, and each of his parents. The control subject was a healthy male, aged 28 years, with normal fertility. Written informed consent was obtained from each participant. This study was approved by the Ethics Committee of the First Affiliated Hospital of Xiamen University.

### Mutations in *DYP19L2* in the patient with globozoospermia

**Fig. 2:**
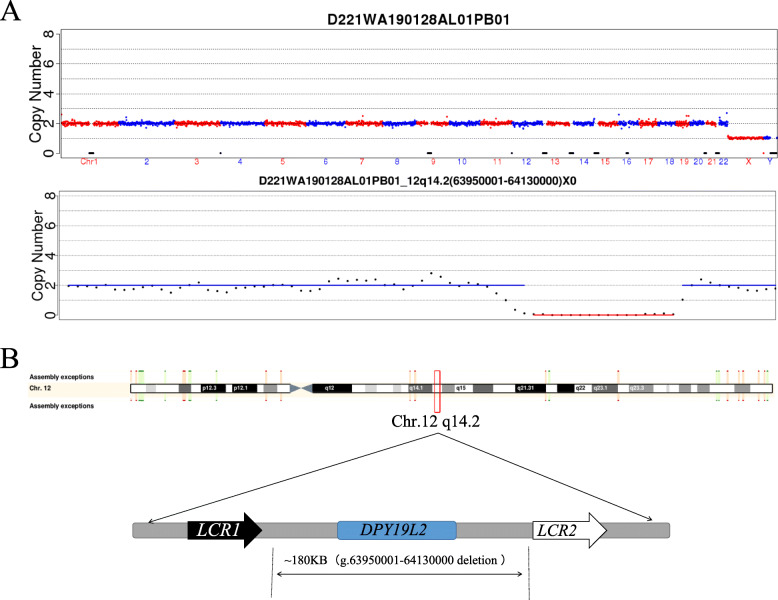
Genome sequencing results of patient samples. **a. The results of CNVseq**: CNV: copy number variation. **b**. **The domains and mutations in*DPY19L2*.** The *DPY19L2* is located on chromosome 12q14.2 and approximately 110.027 kb long. This gene has 22 exons encoding 758 amino acids with a molecular mass of 87.374 kDa. The Chromosome 12q14.2 (g.63950001–64130000) was homozygous deletion of about 180 kb,the mutation leads to complete deletion of *DPY19L2*

In order to determine the genetic variants associated with globozoospermia, we performed copy number variation sequencing (CNVseq) on the DNA samples of the proband and his parents. The mutation was detected in the *DYP19L2* gene (Fig. 1). Approximately 180 kbp of homozygotic deletion was found on chromosome 12 locus 12q14.2 in the patient sample (g.63950001–64,130,000) (Fig. 2a). The functional gene in this segment included in the Decipher database was *DPY19L2*, which is related to spermatogenesis disorder. The main clinical phenotype is round head sperm, which eventually leads to male infertility.

Additionally, the verification by CNV confirmed the homozygous deletion of exons 1, 11, and 22 of *DPY19L2* in the proband. A similar phenotype was detected in the mother’s genome, while no such variation was found in the father’s genome (Supplementary Table 1).

Papanicolaou staining showed that the patient’s sperms had abnormal head development. As shown by the black arrow, the most common sperm defect in the patient was the appearance of the round head (Fig. 1b). Based on these results, the patient was diagnosed with globozoospermia (Fig. 1a, II:1).

Transmission electron microscopy (TEM) further confirmed these defects, and the sperm from the patient showed numerous ultrastructural defects in the head. The ultrastructure of the sperm in the control patient was normal, while that of the proband was round (Fig. 1c).

**Fig. 3:**
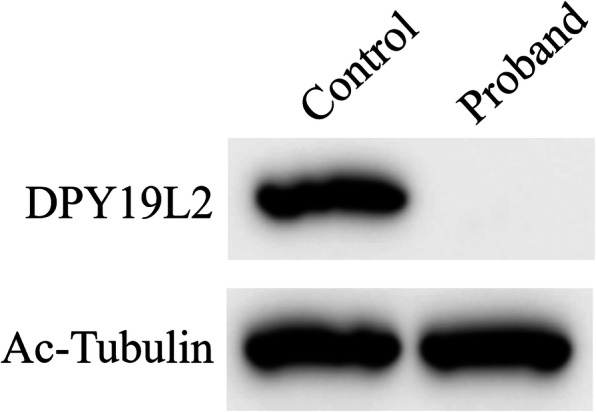
Expression of DPY19L2 in the Patient and normal control. Western blotting analysis of DPY19L2 in sperm cells from control and the proband. Ac-tubulin was used as a loading control

We determined the levels of DYPL92 in the sperm using Western blotting and found that DYP19L2 was not expressed in the sperm of the patient (Fig. 3).

## Discussion and conclusion

Globozoospermia is a disease of sperm malformation characterized by round head sperm and lack of acrosome. These sperms cannot penetrate the zona pellucida of the oocyte, leading to failed fertilization and infertility [29]. When the sperms are injected into the cytoplasm, the fertilization rate tends to be low. Hitherto, the causes of the disorder remain unclear. DPY19L2 is a testis-specific transmembrane protein that is highly expressed in the testes. It anchors the acrosomal membrane to the nuclear membrane and is required for sperm head elongation and acrosome formation during spermatogenesis. However, mutations in this gene lead to instability and loss of acrosome [4, 5, 11, 30]. *DPY19L2* is a pathogenic gene underlying spermatogenic failure, which is an AR inheritance disorder; the homozygous variation might lead to morbidity [31]. In the current study, the data of Papanicolaou staining and electron microscopy provided sufficient evidence of this phenotype in the patient’s sperm. To determine the genetic variation that leads to male infertility, genome-wide CNVseq was performed on the patient presenting globozoospermia. The results showed that *DPY19L2* was absent.

Previous studies have shown that *DPY19L2* mutation types exhibit variations in frequency distribution, which might be attributed to the limited number of patients recruited or analyzed in these studies. Also, the frequency distribution of *DPY19L2* mutation types may be different in pure Chinese patients with this AR genetic disease due to the change in social and cultural factors such as blood relationships [4, 32].

Furthermore, reverse transcription-polymerase chain reaction (RT-PCR) detected the tiny variation of *DPY19L2* in the sample through sequencing; however, the large fragment variation of the gene could not be determined. Thus, other quantitative PCR methods such as MLPA could be utilized to detect the large fragment variation of the gene [4]. Nonetheless, due to the high homology between *DYP19L2* and its pseudogene, selecting a highly specific probe with a unique sequence matching the specific *DPY19L2* nucleotide to overcome the interference of *DPY19L2* pseudogene is essential [4, 32]. Moreover, any loss of heterozygotes shown in MLPA should be further confirmed by a long-range PCR [4].

Herein study, we found that 180-kbp homozygous deletions (g.63950001–64130000) at 12q14.2 on chromosome 12 in the sample of the patient subjected to CNVseq, including complete loss of *DYP19L2*. Complete gene loss leads to complete loss of *DYP19L2*, which might result in complete loss of DYP19L2 function; thus, the sperm of the patient presents globozoospermia. Therefore, this study broadens the mutation spectra of *DYP19L2* mutations that cause globozoospermia.

In summary, our findings confirm that mutations in *DYP19L2* are the major causes of globozoospermia in humans. The homozygosity deletion of *DYP19L2* affects the normal development of sperm head, leading to the typical globozoospermia phenotype. Therefore, the current study provides researchers and clinicians with updated information about sperm with globozoospermia.

## Supplementary information

**Additional file 1: Supplementary Table 1**. Results of exon mutation test on proband and his parents

## Data Availability

The datasets used and/or analyzed during the current study are available from the corresponding author upon reasonable request.

## Abbreviations

TEM: Transmission electron microscopy
AR: Autosomal recessive
RT-PCR: Reverse transcription-polymerase chain reaction
MLPA: Multiplex ligation-dependent probe amplification
CNVseq: Copy number variation sequencing
