# Supplementary material for: Identification of a novel deletion mutation in DPY19L2 from an infertile patient with globozoospermia: a case report
Source: Mol Cytogenet. 2020 Jun 22;13:24. doi: 10.1186/s13039-020-00495-1 (PMC7310204; doi:10.1186/s13039-020-00495-1)
Supplement: Supplementary file 1 — Additional file 1: Supplementary Table 1. Results of exon mutation test on proband and his parents [file 13039_2020_495_MOESM1_ESM.doc]

| Detection results of DPY19L2 (NM_173812.5) | | | | |
| --- | --- | --- | --- | --- |
| Exons | Amino acid mutation | Variation type | Family origin verification | |
|  |  |  | father | mother |
| Exon 1 | - | Homozygous deletion | No mutation | Heterozygous deletion |
| Exon 11 | - | Homozygous deletion | No mutation | Heterozygous deletion |
| Exon 22 | - | Homozygous deletion | No mutation | Heterozygous deletion |

Supplementary Table 1. Results of exon mutation test on proband and his parents
